# The relationship between abdominal fat and change in left ventricular ejection fraction in cancer patients

**DOI:** 10.1002/osp4.454

**Published:** 2020-10-16

**Authors:** Kerryn W. Reding, Khristine Ghemigian, Salvatore Carbone, Ralph D'Agostino, Jennifer H. Jordan, Giselle Meléndez, Zanetta S. Lamar, Heidi D. Klepin, Alexandra Thomas, Dale Langford, Sujethra Vasu, W. Gregory Hundley

**Affiliations:** ^1^ Department of Biobehavioral Nursing and Health Informatics University of Washington School of Nursing Washington Seattle USA; ^2^ Division of Public Health Sciences Fred Hutchinson Cancer Research Center Washington Seattle USA; ^3^ Department of Internal Medicine Section on Cardiovascular Medicine Wake Forest University Winston‐Salem North Carolina USA; ^4^ Department of Internal Medicine VCU Pauley Heart Center Virginia Commonwealth University School of Medicine Richmound Virginia USA; ^5^ Department of Biostatistical Sciences Wake Forest University Winston‐Salem North Carolina USA; ^6^ Department of Biomedical Engineering Virginia Commonwealth University Richmound Virginia USA; ^7^ Department of Pathology Section on Comparative Medicine Wake Forest University Winston‐Salem North Carolina USA; ^8^ Department of Internal Medicine Section on Hematology and Oncology Wake Forest University Winston‐Salem North Carolina USA; ^9^ Department of Anesthesiology and Pain Medicine University of Washington School of Medicine Washington Seattle USA

**Keywords:** body composition, visceral fat

## Abstract

**Objectives:**

Prior studies have identified a relationship between body mass index (BMI) and intraperitoneal (IP) fat with heart failure; however, in prior studies of cancer patients receiving potentially cardiotoxic chemotherapy, elevations in BMI have not necessarily been associated with decrements in heart function. This study tested the hypothesis that IP fat may be associated with left ventricular ejection fraction (LVEF) decline among cancer patients receiving potentially cardiotoxic chemotherapy.

**Methods:**

In this prospective study of 61 cancer patients (23 breast cancer, 32 lymphoma, and 6 sarcoma), IP fat and other assessments of body composition, and changes in LVEF from pre‐ to postcancer treatment using noninvasive magnetic resonance imaging was ascertained.

**Results:**

After accounting for age, baseline LVEF, and confounding variables, pre‐ to 24‐month post‐treatment LVEF changes were inversely correlated with IP fat (*r* = −0.33; *p* = 0.02) and positively correlated with measures of subcutaneous (SQ) fat (*r* = 0.33; *p* = 0.01). These LVEF changes were not correlated with BMI (*r* = 0.12; *p* = 0.37).

**Conclusion:**

Among patients receiving potentially cardiotoxic chemotherapy, pretreatment IP fat was associated with subsequent declines in LVEF. There was no association between BMI and LVEF decline. These findings may be related to a potential protective effect of SQ fat.

## 1 INTRODUCTION

Obesity is defined not by excess weight but by excessive fat accumulation, and is an independent risk factor for cardiovascular diseases (CVDs), including heart failure.^1^ While many studies employ body mass index (BMI) as a proxy for obesity, it is an imprecise measure of adiposity.^2^ This may not be problematic in the general population where BMI remains a strong predictor for CVD^3^; however, in individuals with chronic diseases, including cancer, it may be a less satisfactory metric.^4^, ^5^ One reason is that BMI does not differentiate different body composition compartments, such as fat mass and skeletal muscle mass.^2^, ^6^, ^7^ As a result, in cancer survivors, in whom sarcopenic obesity may occur in response to cancer treatment, BMI may be a particularly poor measure of adiposity.^7^, ^8^, ^9^ Nor does BMI discriminate the location of adiposity. While measures of abdominal adiposity, such as waist to hip ratio, typically provide an improved risk stratification compared to BMI alone,^10^ further improvement may be gained through deep phenotyping of adiposity as differing fat depots convey disparate physiologic impacts depending on anatomical location.^11^ This has been well established, as visceral adipose tissue (VAT) is consistently linked with increased cardiometabolic risk, in contrast with a lack of association or potentially reduced risk with subcutaneous (SQ) fat.^11^, ^12^, ^13^ Still more precision can be gained from separating VAT into its components of intraperitoneal (IP) and retroperitoneal (RP) fat.^14^ IP fat, which contains blood high in lipids potentially due to its location near the hepatic portal vein,^15^ is of particular interest because it is an independent predictor of cardiometabolic risk.^16^

While the distinction of anatomical distribution of adiposity has been appreciated for some time in the cardiovascular literature,^10^ it has received less attention in studies of cancer patients focused on CVD. With CVD being a primary contributor to morbidity and mortality in cancer survivors treated with potentially cardiotoxic chemotherapy, studies have sought to better understand the role of patient risk factors.^17^ Some have shown that BMI modifies the effect of anthracyclines on left ventricular ejection fraction (LVEF),^18^, ^19^ a marker often used to define cardiotoxicity,^20^, ^21^ while others have not.^22^, ^23^, ^24^, ^25^, ^26^, ^27^, ^28^ A potential reason for the inconsistent findings is the imprecision with which BMI approximates obesity and the varying sample sizes of the studies. The goal of this analysis was to investigate the relationships between obesity, IP fat, and LV dysfunction associated with the administration of potentially cardiotoxic chemotherapy through the assessment of postdiagnosis LVEF decline. As such, assessing IP fat as well as BMI may provide the information necessary to help identify the relationship between obesity and LVEF decline at 24‐month postdiagnosis.

## 2 METHODS

### 2.1 Patient population

This prospective study enrolled patients scheduled to receive potentially cardiotoxic chemotherapy from the Wake Forest Comprehensive Cancer Center clinics from January 2013 through February 2016. Eligibility criteria included age >21 years, a life expectancy of >2 years, and a treatment protocol including receipt of potentially cardiotoxic chemotherapy for breast cancer (stages I–III), lymphoma, or soft tissue sarcoma, which included patients scheduled to receive one or more of the following: anthracycline, cyclophosphamide, taxane, or trastuzumab. Patients with contraindications to a cardiovascular magnetic resonance (CMR) exam (e.g., implanted electronic devices) were excluded. In total, 213 consecutive patients were screened for eligibility. Of those, 175 were deemed potentially eligible; of those, 116 were confirmed to be eligible. Of the 116 eligible patients, 54 did not participate in this study: 39 declined to participate (either in the study as a whole or in the abdominal magnetic resonance imaging [MRI] exam) for the reasons of too busy (*n* = 13), no interest or uncomfortable with nature of the study (*n* = 12), none given (*n* = 14); 12 were not enrolled due to the study being unable to accommodate study visits prior to treatment; and three for other reasons. One participant was lost to follow‐up.

CMR images to assess LVEF were acquired at baseline (i.e., prior to the first cycle of chemotherapy) and at 24 months postbaseline. In addition, at baseline body composition was ascertained using measures of height, weight, and abdominal MRI measures of intra‐abdominal and SQ fat. This study was approved by the Wake Forest Health Sciences Institutional Review Board and all participants provided written, witnessed informed consent.

### 2.2 CMR analysis

To measure LVEF, images were acquired using a 1.5 Tesla Avanto (Siemens Healthcare) MRI scanner. MR imaging was chosen to assess LVEF due to its accuracy and prior use in US National Institutes of Health‐funded initiatives such as the Multi‐Ethnic Study of Atherosclerosis (MESA).^29^ LVEF measurements were obtained using previously published methods,^30^ that included cine bright blood steady‐state free precision techniques with 160 × 120 matrix, a 42 cm field of view, an 8‐mm‐thick slice with a 2‐mm inter‐slice gap, and a 33‐ms temporal resolution.

The CMR cine slices were manually analyzed using QMASS (Medis) for the purpose of determining LV volumes and ejection fraction. A reader blinded to the patient and visit information manually outlined the endocardium and epicardium from the end‐diastolic and end‐systolic phases for the baseline and 24‐month visits. The end‐diastolic and end‐systolic volumes as well as the LVEF were calculated according to modified Simpson's Rule Technique from manual contours.^31^

### 2.3 Analysis of abdominal SQ fat and VAT

Using the 1.5 Tesla Avanto MRI scanner, abdominal scans were performed according to previously published techniques.^32^ Total and compartmental amounts of abdominal fat were determined from the axial slice positioned at the level of the second lumbar vertebra (L2) with a 256 × 256 matrix, a 5‐mm‐thick slice, a bandwidth of 305 Hertz/pixel, and a field of view to encompass all of the abdomen.

**FIGURE 1:**
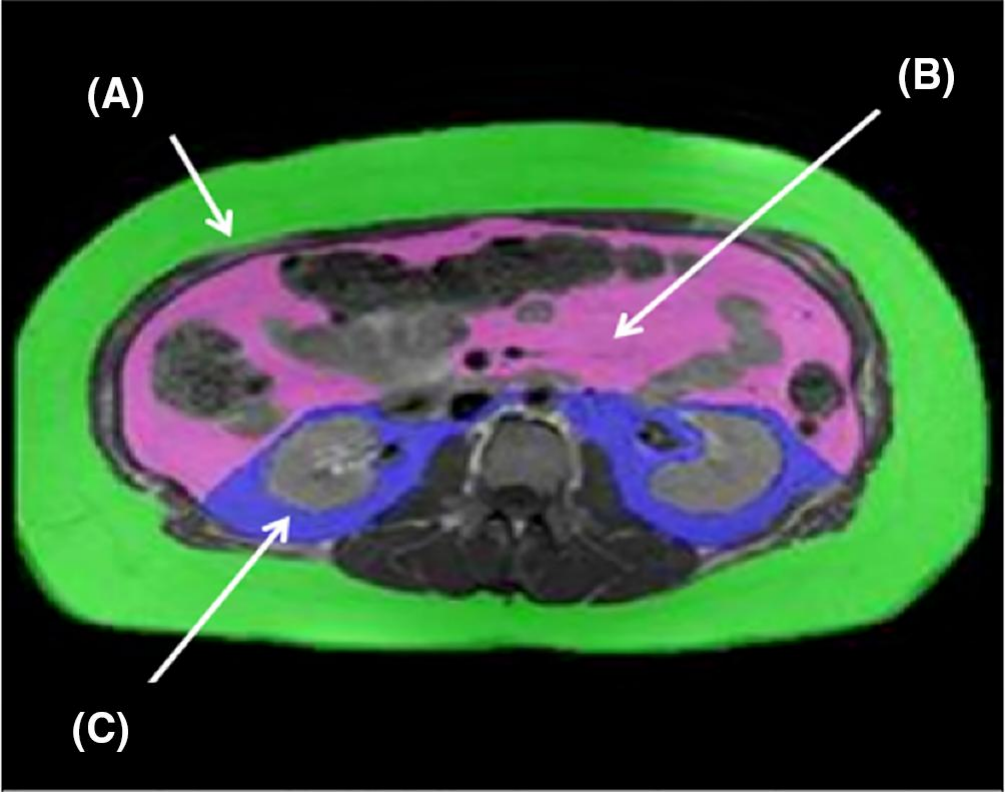
Identification of abdominal fat deposits Images acquired with magnetic resonance at lumbar vertebrate L2 and separated into distinct fat deposits. SQ fat shown as (A), IP fat shown as (B), and RP fat shown as (C). IP, intraperitoneal; L2, second lumbar vertebra; RP, retroperitoneal; SQ, subcutaneous fat

Adipose tissues were segmented and colored from other tissues based on pixel intensity and known divisions of tissue planes using a two phase approach. First, tissue segmentation was performed by an automated algorithm of the SliceOmatic5.0 Rev‐4b2 software program (Tomovision). Second, the blinded reader corrected any misidentified fat or nonfat regions using manual tools provided within the software. To calculate each of the compartmental fat deposits, the number of subpixels within each fat compartment (SQ, VAT, IP, and RP) was multiplied by the individual pixel dimensions within the image and by the slice thickness to determine the area of fat (in cm^2^) for each compartment. Fat depots were separated into abdominal SQ fat and VAT, defined as the fat outside the muscular abdominal wall and the fat to the interior of the abdominal wall, respectively. VAT was further segmented into IP and RP; IP fat was defined as the portion of VAT within the mesentery and omentum bounded anteriorly and laterally by the abdominal wall and posteriorly by a curved line drawn between the kidneys; RP fat as the remaining fat (Figure 1).^33^ Because this analysis focused on depots of body fat, an eligibility criteria for this analysis was the presence of an abdominal MRI; thus, there are no missing data on body fat compartments.

### 2.4 Statistical analysis

For descriptive statistics, continuous variables were presented as means ± standard deviations (SDs), and categorical variables are presented as counts and percentages. Pearson correlation coefficients were estimated to examine the correlations between fat depots and 24‐month LVEF. The analysis examined these correlations graphically by constructing scatter plots between the 24‐month change in LVEF and baseline IP, SQ, and RP fat depots and baseline BMI. The analysis examined the correlation between the 24‐month change in LVEF and measures of baseline VAT, IP, RP, and SQ fat depots and BMI, as unadjusted and adjusted correlations.

For adjustment of confounders, variables were considered that a priori were considered to be a risk factor for LVEF decline. In addition, the variable “time between visits” was used to account for varying time from baseline to the 24‐month visit (for which the mean timeframe for the 24‐month visit was 24.72 months from baseline [interquartile range: 24.12–24.96]) and was included in all models. Thus, our multivariable model included age (in years), gender (male or female), baseline LVEF, receipt of anthracyclines (yes or no), cancer site (sarcoma, lymphoma, or breast cancer), cardiovascular risk factors (a summary variable of hypertension, diabetes and coronary artery disease [CAD]), time between visits, as well as adjustment for IP fat when investigating SQ fat and adjustment for SQ when investigating VAT, IP, and RP fat.

Next, the impact of the IP fat and SQ fat on the 24‐month LVEF change was examined using a general linear model (GLM) adjusted for multivariable model described above. Then the relationship of IP fat and SQ fat with a 24‐month drop in LVEF of 5%, equivalent to the mean decline in LVEF, was examined using logistic regression and a receiver operator curve (ROC) analysis. Secondarily, a LVEF decline was modeled for a commonly used threshold of either a 10% decline from pre‐ to post‐treatment or a decline to 50%.^21^ To assess the overall model fit, the likelihood ratio test was used. A multivariable model was fit using the same set of adjustment variables described above. Then the depots of IP fat and SQ fat were added to this model. To compare one model to another a nonparametric test developed by DeLong et al. was used.^34^

Lastly, exploratory analyses were conducted using a variable that combined IP and SQ fat depots modeled as a ratio of IP: SQ fat. This variable was used as the independent variable of the GLM models, and selected a parsimonious model of adjustment (i.e., those significantly associated with LVEF decline which included baseline LVEF and anthracycline use) plus age, gender, and time between visits. All statistical analyses were performed using SAS (SAS Institute).

## 3 RESULTS

In this dataset of cancer survivors, participants had a mean age of 53.37 years (SD: 15.25), mean weight of 86.11 kg (SD: 17.87) and a mean BMI of 30.14 kg/m^2^ (SD: 5.73; Table 1). Nearly one‐third of our sample was men and approximately three‐quarters were Caucasian. Only four (6.56%) had CAD, 36.07% had hypertension, 11.48% were current smokers, and 16.39% had Type II diabetes. With respect to cancer, 37.70% had been diagnosed with breast cancer, 52.46% with lymphoma, and 9.84% with sarcoma. A majority of these cancer survivors had received the anthracycline doxorubicin (70.49%), and cyclophosphamide (67.21%), while fewer received docetaxel (26.23%), or paclitaxel (4.92%). Among those receiving doxorubicin for breast cancer, lymphoma, and sarcoma, the mean doses (mg) were 439.76 (SD: 75.06), 493.25 (SD: 178.09), and 654.38 (SD: 470.76), respectively. In patients with high body surface area (BSA), dosages did not exceed 250.00 mg/m^2^ in breast cancer patients or 315.00 mg/m^2^ in lymphoma patients. There was no indication in any patient that concerns regarding cardiotoxicity led to treatment interruption. With regards to abdominal adipose tissue depots at baseline, the mean areas of SQ, IP, RP, and VAT were 245.70 cm^2^ (SD: 116.94), 114.86 cm^2^ (SD: 72.63), 54.38 cm^2^ (SD: 37.02), and 169.24 cm^2^ (SD: 102.47), respectively. There was a significant decline in LVEF over 24‐month (*p* < 0.001) from a mean at baseline of 62.09% (SD: 8.10) to a 24‐month mean of 56.10% (SD: 9.71) for a mean LVEF decline of 5.00% (SD: 8.17).

**TABLE 1:** Patient characteristics

	Study participants (*N* = 61)^a^ ^,^ ^b^
Demographics	
Age (years)	53.37 ± 15
Male	19 (31.15%)
Caucasian	47 (77.05%)
Height (cm)	169.49 ± 10.60
Weight (kg)	86.11 ± 17.87
BMI (kg/m^2^)	30.14 ± 5.73
CVD risk factors and outcomes	
Systolic BP	118.90 ± 17.53
Diastolic BP	69.83 ± 13.47
Heart rate (bpm)	72.63 ± 12.74
Current smoker	7 (11.48%)
Hypertension	22 (36.07%)
Diabetes	10 (16.39%)
Coronary artery disease	4 (6.56%)
Current medications	
ACE inhibitor	9 (14.75%)
ARB	4 (6.56%)
Aspirin	19 (36%)
Beta blocker	4 (6.56%)
Calcium channel blocker	5 (8.20%)
Cancer type	
Breast	23 (37.70%)
Lymphoma	32 (52.46%)
Sarcoma	6 (9.84%)
Chemotherapy	
Doxorubicin	43 (70.49%)
Paclitaxel	3 (4.92%)
Docetaxel	16 (26.23%)
Cyclophosphamide	41 (67.21%)
Therapy with monoclonal antibodies	
Trastuzumab	1 (1.64%)
Rituximab	18 (29.51%)
Depots of fat (cm^2^)	
Subcutaneous	245.70 ± 116.94
Intraperitoneal	114.86 ± 72.63
Retroperitoneal	54.38 ± 37.02
Visceral (IP + RP)	169.24 ± 102.47
Left ventricular characteristics	
End diastolic volume, baseline (ml)	125.47 ± 33.51
End diastolic volume, 24‐month (ml)	123.50 ± 40.07
End systolic volume, baseline (ml)	48.44 ± 19.12
End systolic volume, 24‐month (ml)	54.12 ± 23.66
Ejection fraction, baseline (%)	62.02 ± 8.05
Ejection fraction, 24‐month (%)	56.80 ± 9.90

Abbreviations: ACE, angiotensin converting enzyme; ARB, angiotensin receptor blocker; BMI, body mass index; BP, blood pressure; CVD, cardiovascular disease; IP, intraperitoneal; RP, retroperitoneal.

^a^ Mean (standard deviation) for continuous variables; *n* (%) for categorical variables.

^b^ No missing data for any variables.

**FIGURE 2:**
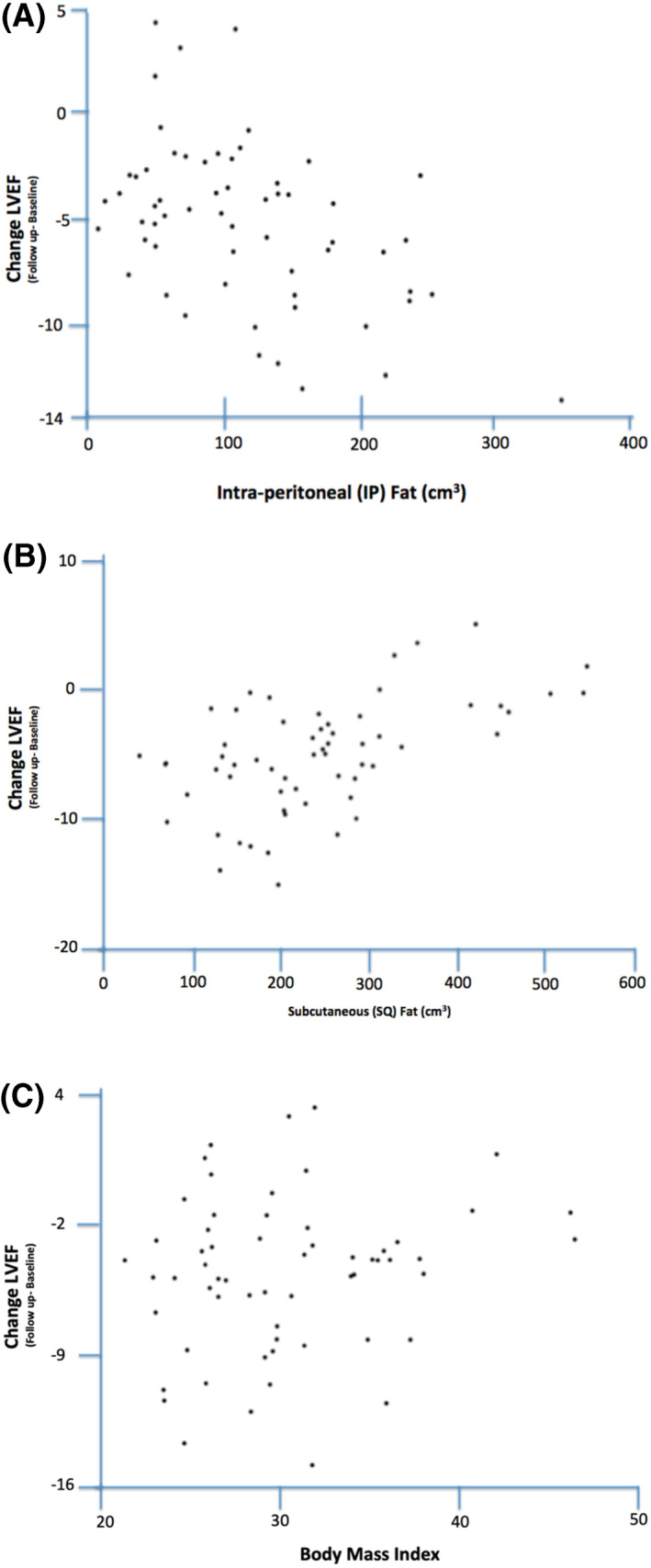
Correlations between 24‐month LVEF change and body composition. Change in LVEF compared to baseline amount of and IP (A), SQ (B), and BMI (C), when adjusted for baseline ejection fraction, gender, time, anthracycline use, and height (SQ and IP only). AUC, area under the curve; BMI, body mass index; CVD, cardiovascular disease; IP, intraperitoneal; LVEF, left ventricular ejection fraction; SQ, subcutaneous fat

While BMI was not correlated with changes in LVEF over 24 months of follow‐up (*r* = 0.09; *p* = 0.50, *r* = 0.12; *p* = 0.37 in unadjusted and adjusted analyses, respectively), both IP fat and SQ fat were (Figure 2). IP fat at baseline was inversely correlated with change in LVEF at 24‐month (*r* = −0.33; *p* = 0.02) while SQ fat was positively correlated with 24‐month LVEF change (*r* = 0.37; *p* = 0.01), after adjustment for confounders. VAT was marginally inversely correlated with LVEF change (*r* = −0.27; *p* = 0.05) and no correlation was observed between RP fat and LVEF change (*r* = −0.09; *p* = 0.54) in either the adjusted or unadjusted analysis (Table 2).

**TABLE 2:** Correlations between baseline depots of body fat and change in LVEF at 24‐month

	*r* (unadjusted)	*p*‐value	*r* (adjusted^a^)	*p*‐value
Depot of fat^b^				
VAT	−0.17	0.18	−0.27	0.05
IP	−0.21	0.11	−0.33	0.02
RP	−0.08	0.55	−0.09	0.54
SQ	0.31	0.01	0.37	0.01

Abbreviations: IP, intraperitoneal; LVEF, left ventricular ejection fraction; RP, retroperitoneal; SQ, subcutaneous; VAT, visceral adipose tissue.

^a^ For the VAT, IP, and RP variables, adjusted for age, gender, baseline LVEF, anthracycline use, CVD risk factors, cancer site, time between visits, and SQ fat. For the SQ variable, adjusted for the same factors, with the exception of IP fat (rather than SQ fat).

^b^ No missing data for these variables.

Table 3 displays all factors in the multivariable model, showing that anthracycline use and baseline LVEF (*p* = 0.02 each) were in addition to IP fat and SQ fat (*p* = 0.02 and 0.01, respectively) significantly associated with LVEF change. Using estimates from the GLM, the LVEF change was computed for differing levels of IP and SQ fat so that contrasting levels of IP and SQ fat could be modeled in two patients for whom the only difference would be their levels of IP and SQ fat. This fictitious set of two patients were modeled as male, 50‐year old patients with a baseline LVEF of 62.02% (the mean of our sample) and a diagnosis of lymphoma for which they were treated with anthracyclines. The first patient had low IP fat (calculated as the mean—1 SD = 42.23 cm^2^) and a high SQ fat (mean + 1 SD = 362.64 cm^2^) whose resulting decline in LVEF equaled 4.47. The second patient had high IP (mean + 1 SD = 187.49) and low SQ (mean—1 SD = 128.76) whose resulting decline in LVEF equaled 15.68.

**TABLE 3:** Relationships between risk factors and LVEF change at 24‐month in patients treated with potentially cardiotoxic chemotherapies

Variable^a^	β	*SE*	*p*‐value
Intercept	1.92	11.46	0.89
Time between visits	0.02	0.01	0.08
Baseline LVEF	−0.28	0.12	0.02
Receipt of anthracycline	−6.27	2.65	0.02
Female gender	−2.99	2.56	0.25
Age (years)	−0.09	0.07	0.19
CVD risk factors	−1.69	1.18	0.16
Breast cancer	−3.84	2.77	0.17
SQ fat^b^	0.03	0.01	0.01
IP fat^b^	−0.04	0.01	0.02

Abbreviations: CVD, cardiovascular disease; IP, intraperitoneal; LVEF, left ventricular ejection fraction; SE, standard error; SQ, subcutaneous.

^a^ Generalized linear models showing all variables in the model; nonitalicized are putative risk factors.

^b^ Unadjusted *p*‐value for SQ fat was 0.02; for IP fat was 0.09.

**FIGURE 3:**
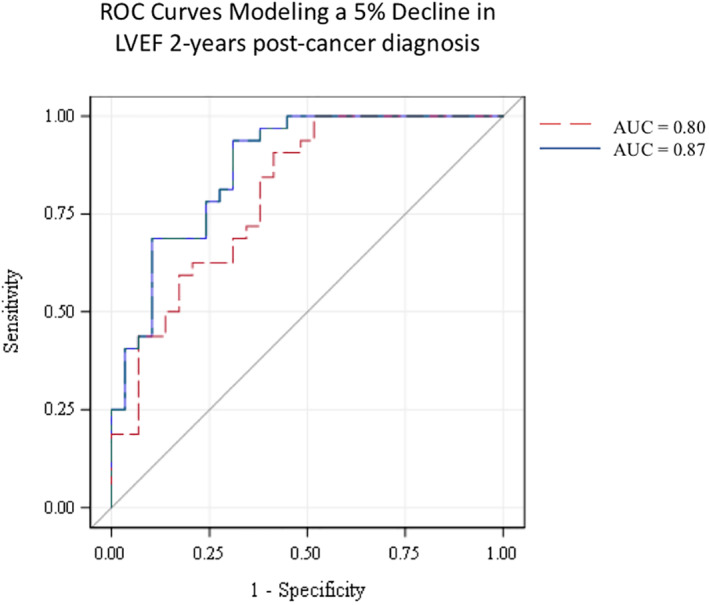
Receiver operator characteristic curves modeling 2‐year LVEF decline of 5%. The red dashed line represents a model incorporating age at diagnosis, gender, baseline LVEF, anthracycline use, CVD risk factors, cancer site, and time between visits (AUC = 0.80). The blue line represents the above model plus the addition of IP and SQ depots of fat, demonstrating the improvement in AUC when the IP fat and SQ fat added to the ROC curve (AUC = 0.87). AUC, area under the curve; CVD, cardiovascular disease; IP, intraperitoneal; LVEF, left ventricular ejection fraction; ROC, receiver operator characteristic

In a model designed to forecast a 2‐year LVEF decline of 5%, the area under the curve (AUC) for the multivariable model without IP fat or SQ fat (i.e., age, gender, baseline LVEF, anthracycline use, CVD risk factors, cancer site, and time between visits) was 0.80. Adding IP fat and SQ fat to this multivariable model raised the AUC to 0.87 (Figure 3). The *p*‐values for the IP fat and SQ fat terms in this model were 0.03 and 0.02, respectively, and the *p*‐value comparing the incremental improvement to the ROC curve by addition of IP fat and SQ fat was trending toward statistical significance (*p* = 0.08). The ROC curve was further assessed by modeling a commonly used threshold for cardiac dysfunction (of 10% LVEF decline or a decline to 50% LVEF postdiagnosis). Here the inclusion of IP fat and SQ fat (*p*‐value of 0.10 and 0.05, respectively) changed the AUC from 0.80 to 0.83 comparing the incremental improvement from a multivariable model without versus with IP fat and SQ fat.

Lastly, exploratory analysis was performed investigating the joint effects of baseline IP and SQ fat depots. The ratio of IP:SQ fat was not statistically significantly correlated with change in LVEF at 24‐month (*r* = −0.21; *p* = 0.13).

## 4 DISCUSSION

In this study among patients treated with potentially cardiotoxic treatment, LVEF decline at 2 years was inversely correlated with abdominal fat in the IP region of the viscera and positively correlated with SQ fat at cancer diagnosis. This is contrasted with our finding that BMI, which does not differentiate body fat distribution, was not associated with 24‐month postchemotherapy declines in LVEF. In addition, our data suggest that addition of prediagnostic IP and SQ fat depots improves the ability to predict LVEF decline 2 years after cancer diagnosis. While research in cancer survivors has investigated relationships between body composition and survival, to our knowledge none have investigated relationships between depots of fat and LVEF decline. In a population of cancer survivors receiving potentially cardiotoxic treatment, this analysis evaluated combinations of regional fat depots as risk factors for LVEF decline.

Our results showed no correlation between BMI and change in LVEF. This finding in combination with our observation that IP and SQ fat depots were associated with a 2‐year decline in LVEF suggests that body fat distribution may play a more prominent role than BMI in determining cardiac function decline over time after receipt of potentially cardiotoxic chemotherapy. More specifically, the lack of association with BMI may be due to the protective effect seen with SQ fat. Our findings suggesting opposing effects of IP and SQ fat are consistent with prior work in noncancer populations showing an increased cardiometabolic risk with VAT and a reduced risk with SQ fat.^12^

BMI has been investigated in relation to cardiotoxicity based on its role in the development of CVD and due to the use of BSA as a factor guiding chemotherapy dosing.^35^, ^36^, ^37^ Our findings of a lack of association with BMI and LVEF decline differ from those in a 2016 meta‐analysis involving breast cancer patients who were treated with anthracyclines alone or sequential anthracyclines and trastuzumab.^35^ While the meta‐analysis showed a 1.38‐fold increased risk of cardiotoxicity associated with a BMI ≥25 kg/m^2^, the meta‐analysis study design did not allow for control of obesity‐related cardiovascular risks factors such as diabetes and hypertension, thus leaving the possibility that unmeasured confounding by cardiovascular risk factors were responsible for the elevated risk. Moreover, methodologic issues have been raised pertaining to the pooling of studies with different designs, sample sizes, and definitions of cardiotoxicity that could influence the overall study results.^38^ Our analysis, by contrast, used precise CMR‐derived measures of LVEF, and would not necessarily suffer from this concern.

These findings showed relationships of IP fat and SQ fat with LVEF decline in opposing directions. For interpretability, two sample patients were modeled with contrasting IP and SQ fat depots, showing that a patient with low IP and high SQ fat had a LVEF decline less than 5%, the mean decline in cancer patients. This was contrasted with a patient with high IP and low SQ whose LVEF decline equaled 15.68, corresponding to a 24‐month LVEF of 46.34%, which exceeds a commonly used threshold of LVEF decline for cardiotoxicity.^20^, ^21^

This concept of a body composition phenotype of high visceral fat with low SQ fat is well described in the literature.^11^, ^13^, ^39^, ^40^ Mechanistically, in the presence of excessive adipose accumulation, SQ fat may be redirected to ectopic fat storage, including in the IP region.^11^, ^39^ When the SQ fat depot is overwhelmed by an overabundance of lipids, it shunts lipids away from SQ to a visceral placement of adipose tissue, thereby creating a phenotype of low SQ in the presence of high VAT.^10^ The implications of this are described in an extensive set of literature showing that an accumulation of VAT, particularly in the IP region, is associated with increased cardiometabolic risk.^13^, ^15^, ^16^ It is hypothesized that the anatomical location of IP fat (which contains the mesenteric fat) near the portal vein may be primarily responsible for its worse impacts on health.^39^ This is supported by studies showing a higher lipid content may be driving differing gene expression in IP fat versus SQ fat.^41^ In particular, IP fat produces a number of proinflammatory cytokines, such as interleukin‐1, associated with adverse cardiac remodeling contributing to an increased risk of cardiac dysfunction reported in patients with obesity.^42^

Within the cancer literature, Nattenmuller et al. reported increased baseline VAT:SQ fat and not BMI was associated with reduced survival in lung cancer patients.^43^ This analysis showed that an increase in VAT during treatment did not translate to statistically significant increases in BMI due to a significant loss of muscle mass, further supporting the concept that BMI is not sensitive enough to detect changes in body composition, at least in cancer patients. Similarly, two recent studies in nonmetastatic breast cancer and colorectal cancer patients have shown that measures of body composition such as adiposity and muscle mass outperform BMI in determining cardiovascular risk and survival.^4^, ^5^ Additionally, it was recently shown in a large cohort of breast cancer survivors that baseline VAT was associated with a 1.2‐fold increased CVD risk,^44^ which is aligned with our study's findings of an increased risk of LVEF decline in relation to elevated baseline VAT. However, this recent study did not investigate whether LVEF decline was a mechanism by which CVD risk was increased, nor did it investigate deep phenotyping of abdominal fat focused on the IP portion of VAT.

Finally, inclusion of IP and SQ fat depots at baseline significantly predicted LVEF decline 2 years after cancer diagnosis. When baseline IP and SQ fat depots were added to a 7‐variable model, including CVD risk factors, receipt of anthracycline, and LVEF prior to cancer treatment, to predict 2‐year LVEF decline, the AUC was elevated from 0.80 to 0.87. This suggests that this increase in predictive ability is due to the discernment of fat distribution phenotypes that the IP and SQ fat depots provide, and suggests that SQ and IP fat depots need to be considered in the context of one another, such that together they provide a more complete picture of adipose storage.

This study used abdominal MRI to conduct deep phenotyping of body adiposity. MRI, the only nonradiological technique for quantifying depots of body adiposity, has been validated by our group and others in the assessment of IP and SQ fat at the L2 vertebrae.^32^, ^33^ In the same set of patients, CMR was used to detect cardiac changes postchemotherapy. CMR, a gold standard for the assessment of LVEF,^45^ was used to measure LVEF prior to and 24‐month after initiating cancer treatment. Moreover, by utilizing CMR, which is highly sensitive to LVEF changes, our study was able to more precisely detect changes in LVEF than can be reliably assessed in other methods, such as echocardiogram. As a result, associations with a 5% decline in LVEF could be identified, and then following up this finding, our analysis investigated predictors of a 5% LVEF decline in the ROC curves.

Our study had limitations. First, our study population included patients from a single institution with a variety of cancers treated with a variety of anti‐neoplastic regimens. However, the fact that different cancer types and treatments were included in the analysis broadens the applicability of our findings. Despite this, the findings may not be generalizable, particularly to cancer patients who do not receive potentially cardiotoxic treatments. Second, our study does not have muscle mass quantified at this time. Thus, this study is unable to include a comparison of lean to fat mass within this analysis. Lastly, this study is not able to investigate the effects of temporal changes in fat depots during cancer treatment on change in LVEF. Serial measurements of these fat depots throughout a patient's cancer treatment could help determine if relative changes in regional fat depots during treatment are related to changes in LVEF.

## 5 CONCLUSIONS

Our data suggests that assessment of locations of adipose storage is useful in understanding the relationship between obesity and late effects of potentially cardiotoxic cancer therapy. This could be due to the additional information provided by the comparison of SQ fat to ectopic fat storage in the IP region of the viscera. While additional studies are needed to replicate these findings prior to these results being actionable in a clinical setting, the physiologic basis of our findings are supported by the literature in obesity and CVD that point to a role of elevated IP in relation to reduced SQ fat.^13^, ^15^, ^16^ However, given the challenge of measuring depots of fat through imaging on all patients, the development of predictive statistical models to estimate IP and SQ fat depots, such as those developed in adolescents,^46^ may provide a tool for identifying people with elevated IP fat in relation to SQ. Overall, this line of work could identify patients at greatest risk for LVEF decline, and ultimately lead to interventions aimed at reducing excess IP fat. With exercise training and caloric restriction shown to reduce VAT,^47^, ^48^ such an intervention could be employed as a potential therapeutic strategy for the prevention of LVEF decline in this population.
